# Interactive relations between maternal prenatal stress, fetal brain connectivity, and gestational age at delivery

**DOI:** 10.1038/s41386-021-01066-7

**Published:** 2021-06-29

**Authors:** Moriah E. Thomason, Jasmine L. Hect, Rebecca Waller, Paul Curtin

**Affiliations:** 1 Department of Child and Adolescent Psychiatry, New York University Medical Center, New York, NY USA; 2 Department of Population Health, New York University Medical Center, New York, NY USA; 3 Neuroscience Institute, NYU Langone Health, New York, NY USA; 4 Medical Scientist Training Program, University of Pittsburgh & Carnegie Mellon University, Pittsburgh, PA USA; 5 Department of Psychology, University of Pennsylvania, Philadelphia, PA USA; 6 Department of Environmental Medicine and Public Health, Icahn School of Medicine at Mount Sinai, New York, NY USA

**Keywords:** Risk factors, Neural patterning

## Abstract

Studies reporting significant associations between maternal prenatal stress and child outcomes are frequently confounded by correlates of prenatal stress that influence the postnatal rearing environment. The major objective of this study is to identify whether maternal prenatal stress is associated with variation in human brain functional connectivity prior to birth. We utilized fetal fMRI in 118 fetuses [48 female; mean age 32.9 weeks (*SD* = 3.87)] to evaluate this association and further addressed whether fetal neural differences were related to maternal health behaviors, social support, or birth outcomes. Community detection was used to empirically define networks and enrichment was used to isolate differential within- or between-network connectivity effects. Significance for χ^2^ enrichment was determined by randomly permuting the subject pairing of fetal brain connectivity and maternal stress values 10,000 times. Mixtures modelling was used to test whether fetal neural differences were related to maternal health behaviors, social support, or birth outcomes. Increased maternal prenatal negative affect/stress was associated with alterations in fetal frontoparietal, striatal, and temporoparietal connectivity (β = 0.82, *p* < 0.001). Follow-up analysis demonstrated that these associations were stronger in women with better health behaviors, more positive interpersonal support, and lower overall stress (β = 0.16, *p* = 0.02). Additionally, magnitude of stress-related differences in neural connectivity was marginally correlated with younger gestational age at delivery (β = −0.18, *p* = 0.05). This is the first evidence that negative affect/stress during pregnancy is reflected in functional network differences in the human brain in utero, and also provides information about how positive interpersonal and health behaviors could mitigate prenatal brain programming.

## Introduction

Children born to mothers who endure heightened psychological or physiological stress during pregnancy may experience negative consequences as a result of these early programming events. Injurious effects of prenatal stress can cut across domains, increasing risk for neuropsychiatric and neurodevelopmental disorders [1–4], altering hormonal [5–7] and physiological [8–10] bodily responses, and increasing susceptibility to a range of disease processes [11–14]. Studies of fetal behavior and physiology suggest that maternal prenatal stress may influence fetal brain development even before birth [15–17]. Furthermore, negative consequences of early stress exposure appear to be lasting, affecting even the prevalence of adult neurodegenerative disorders [18] thus shaping long-term outcomes and potentially contributing to the transfer of risk to subsequent generations [19, 20].

A long-standing challenge for this crucial area of research is that children born to mothers exposed to high levels of prenatal stress experience more birth complications [21], and are more frequently reared in high-stress environments [22], confounding our ability to conclude from postnatal brain measurements that the fetal brain is altered before birth. Animal studies show that prenatal stress results in shorter and less complex dendrites, hypomyelination, and altered synaptogenesis [23–26]. Postnatal studies in neonates and children corroborate these observations showing that stress alters newborn functional neural connectivity [27–29] and child structural brain development [30]. Importantly, differences in child brain structure have been shown to mediate, in part, the association between prenatal stress and affective problems in childhood [31]. While such findings suggest that prenatal stress alters the fetal brain, examining these outcomes postnatally represents a major limitation. Prospective evidence that the fetal brain is altered is needed to provide stronger evidence that maternal stress during pregnancy impacts the human brain in utero.

The present study addresses this gap by leveraging emergent functional magnetic resonance imaging (fMRI) techniques to evaluate, for the first time, whether and how variation in maternal stress relates to human fetal brain system organization. Resting-state functional connectivity (RSFC) fMRI has recently been adapted to study fetal brain functional network development [32, 33]. This technique relies on recording spontaneous functional signals across the whole brain, and then evaluating covariation and interaction between signals over time. Brain regions that demonstrate coordinated activity are considered “functionally connected”, which has been shown to have an anatomical basis [34–36]. This technique has borne rapid insight into the global coordination of fetal brain activity and the wide scale architecture of brain networks. There is now evidence that RSFC develops initially in utero [37–41], is altered in fetuses with atypical neuroanatomy [42], differs between the sexes [43], relates to infant outcomes [44], and is prospectively diminished in fetuses born preterm [45].

The primary objective of this study is to establish whether maternal prenatal stress relates to changes in the child’s brain before birth; the second is to determine whether psychosocial support and health behaviors affect this association; and the third is to test whether this neurobiological embedding of stress relates to how early a child will be born. There is limited data on normative processes of fetal brain functional network development, as such, our hypotheses are focused on regions central to programming large-scale fetal functional network architecture and commonly implicated in psychiatric and neurodevelopmental diseases [39, 46, 47]. Specifically, we hypothesize that maternal prenatal negative affect/stress (NAS) will be associated with altered connectivity of high-order association cortices, specifically prefrontal and parietal regions, and with connectivity of the insular and temporal regions. In addition, because the effects of stress vary widely from person to person, we further hypothesized that higher levels of social support and adaptive health behaviors would moderate the impact of maternal NAS on child prenatal brain development. Finally, motivated by prior work that has linked prenatal stress to premature delivery [48–50], we hypothesized that greater magnitude of association between maternal NAS and fetal brain connectivity effects would relate to younger age at delivery.

## Materials and methods

### Participants

The fetal neuroimaging sample consisted of 118 cases (48 female), with mother mean age 25.1 years (*SD* = 4.5). Exclusions for participation included presence of suspected fetal central nervous system abnormality as determined by 20-week ultrasound and/or contraindication for MRI (e.g., pacemaker, ferromagnetic material in mother’s body, claustrophobia). The mean age of fetuses at the time of MRI was 32.9 weeks GA (*SD* = 3.87; range 26–39 weeks), and mean age at birth was 39.4 weeks GA (*SD* = 1.1). One hundred five cases in this sample have been studied in prior functional connectivity studies [39, 45, 51] and 118 of these cases were studied in a recent investigation of prenatal sex differences [43]. Ultrasound examination administered by a referring physician was performed within 1 week of MRI examination to determine fetal GA. All women provided written informed consent before undergoing MRI examination. Participation was approved by Human Investigation Committee of Wayne State University. Study participants were followed longitudinally to assure that they developed no complications during pregnancy. Sociodemographic characteristics and birth outcomes in mother–child dyads (*N* = 118) are provided in SI Appendix, Table S1 and a summary table for all case exclusions is provided in SI Appendix, Table S2.

### Definition of maternal prenatal stress

**Fig. 1:**
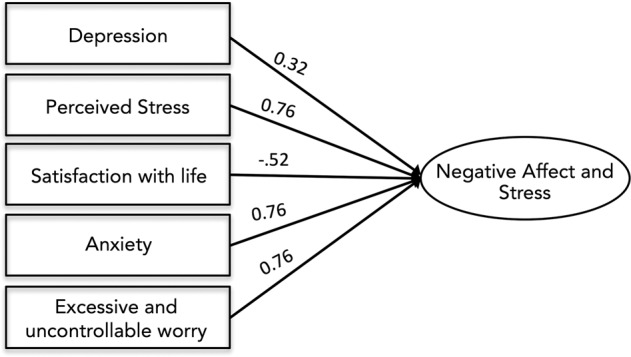
Confirmatory Factor Analytic model showing loadings of stress and internalizing indicators onto prenatal stress and negative affect factor, or NAS. All factor loading scores were significant at <0.001. Model showed excellent fit to the data: χ^2^ = 10.23, *p* = 0.07, df = 5; CFI = 0.98; TLI = 0.96; RMSEA = 0.07; SRMR = 0.03. Measures included the Center for Epidemiological Studies Depression Scale (CES-D), the State Trait Anxiety Inventory (STAI), the Penn State Worry Questionnaire (PSWQ), the Perceived Stress Scale (PSST), and the Satisfaction with Life Scale (SWLS).

Conceptualization and measurement of stress has changed over time [52]. Prior studies of the effects of intrauterine stress have used biological measures such as salivary cortisol and self-report questionnaires of anxiety, depression, and stress [30, 31, 53]. Here, we empirically derived a single factor representing prenatal Negative Affect and Stress (NAS) and in supplementary analyses tested the association of this factor and all subscales with salivary cortisol in a subset of mothers for which cortisol measurements were available. The primary advantage of this approach is reducing the number of statistical tests performed across inherently co-linear measures, while maintaining individual measure loadings [54]. Maternal stress during pregnancy was assessed using summary scores from five scales that assessed internalizing problems and stress: the Center for Epidemiological Studies Depression Scale CES-D; [55] the State Trait Anxiety Inventory (Trait) STAI-T [56]; the Penn State Worry Questionnaire PSWQ [57]; the Perceived Stress Scale PSST [58]; and the Satisfaction with Life Scale SWLS [59]. Descriptive statistics for prenatal affect and stress scales are provided in SI Appendix, Table S3. To account for variance in stress exposure and negative affect, as well as minimize the number of models run, we used factor analysis in in Mplus vs. 7.2 [60] to derive a maternal NAS index. Specifically, in a random half of the sample, we subjected the five scales to exploratory factor analysis (EFA) requesting models with one and two factors. EFA established that these scales loaded best onto a single factor (CFI = .98, TLI = .96, RMSEA = .08, SRMR = .03; all factor loadings, *p* < .001). We next subjected the scales to confirmatory factor analysis (CFA) in the other random half of the sample to validate the model fit. Again, the five scales showed high loadings and good fit to a one-factor model (*n* = 99, CFI = .98, TLI = .97, RMSEA = .06, SRMR = .03; factor loadings, *p* < .001). We thus reran the CFA in the full sample to obtain individual NAS scores within the full sample. The model showed good fit to the data (χ^2^ = 10.23, *p* = 0.07, df = 5; CFI = 0.98; TLI = 0.96; RMSEA = 0.07; SRMR = 0.03) with each scale loading significantly on the NAS factor (*p* < .001). Individual factor loadings are provided in Fig. 1.

### Functional data preprocessing

Time frames corresponding to periods of minimal head motion in the fetus were identified using FSL image viewer [61]. Brainsuite [62] was used to manually generate 3D masks for single reference images drawn from time periods, or segments, of fetal movement quiescence. Masks were binarized and applied only to frames corresponding to their select segment, and only those data were retained for further analyses. Each segment was manually reoriented, realigned to the mean BOLD volume, resampled to 2 mm isotropic voxels, and normalized to a 32-week fetal brain template [63] using affine transformation in Statistical Parametric Mapping (SPM8) [64] software implemented in MATLAB. Motion parameters for each low-motion segment were checked to ensure only segments that consisted of at least 20 sec (10 frames) of low motion were retained in subsequent processing steps. The level of censoring applied, 1 mm mean XYZ and 1.5° mean PYR, has been reported previously [39, 45, 51]. Application of these quality assurance steps resulted in elimination of mean = 56% frames per participant. The resulting sample was 118 cases with an average of 159 frames (SD = 42). Average translational and rotational motion across subjects ranged from 0.01 to 0.52 mm and 0.6° to 1.1°, respectively. In a final step, to correct for variation in normalization across segments within-participant, normalized images were concatenated into one run, realigned to the mean BOLD volume, and smoothed with a 4 mm FWHM Gaussian kernel.

### Functional brain segmentation

A spatially constrained group level clustering approach [65] was used to parcellate the area of a 32-week GA fetal template brain [63] into 197 spatially contiguous, similarly sized ROIs (SI Appendix, Fig. S1). This method utilizes 4D fetal data normalized to 32-week fetal template space and produces functionally homogenous clusters by assessing voxel timeseries similarity in a given dataset, using Pearson correlations, then iteratively merging voxels that showed maximal within-cluster similarity and minimal between-cluster similarity. Next, it identifies the most representative clusters of voxels using a normalized cut algorithm [66] and performs group level clustering. This approach yields ROIs that span the full extent of cortical, subcortical and cerebellar regions and is representative of observed functional connectivity patterns in this fetal sample, after application of motion correction, normalization, and concatenation steps. This is an established data-driven approach to parcellation [39, 43, 67, 68], useful in the human fetal brain where priors are limited and developmental change is exceedingly rapid. ROIs were then classified by hemisphere, by lobe, and by coordinates corresponding to center of mass.

### Derivation of fetal brain networks

**Fig. 2:**
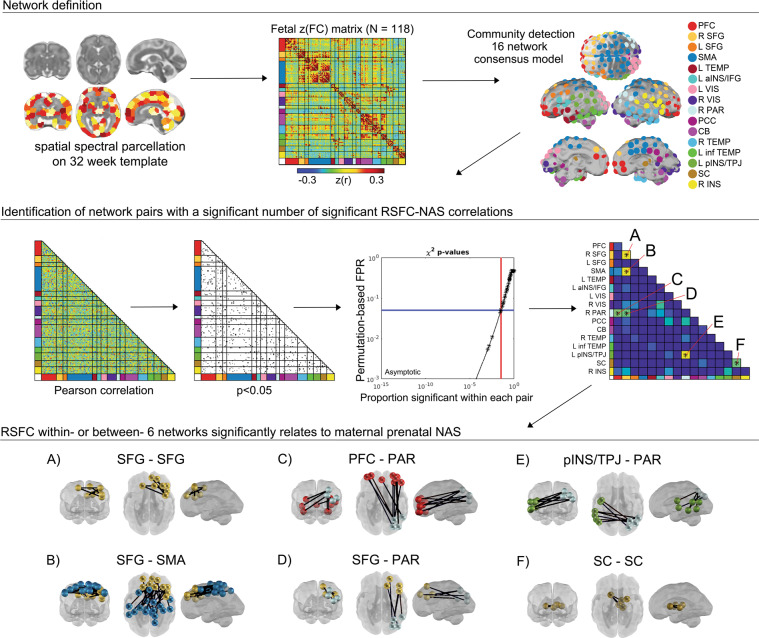
Examination of stress-related variation in fetal brain functional connectivity. Community detection analysis generated a 16 functional network consensus model from a matrix of 197 functionally defined regions. Enrichment was performed to assess the degree to which NAS correlated edges (Pearson *p* < 0.05) in a network pair were overrepresented, tested using a permutation-based estimated false positive rate (FPR). The result was six network pairs with differences in connectivity related to maternal prenatal NAS (**A**–**F**). Within and between-network pairs enriched with significant ROI–ROI resting-state functional connectivity (RSFC) are displayed in the bottom panel, displayed on a 32-week human fetal brain cortical surface. PFC prefrontal cortex, SFG superior frontal gyrus, SMA somatomotor area, aINS anterior insula, IFG inferior frontal gyrus, VIS Visual, PAR parietal, PCC posterior cingulate cortex, CB cerebellum, inf TEMP inferior temporal, pINS posterior insula, TPJ temporoparietal junction, SC subcortical gray matter.

CONN functional connectivity toolbox (v14n) [69] was used to generate Pearson correlations matrices between these 197 ROIs for each subject. Processing included linear detrending, nuisance regression using aCompCor of five principal components extracted from a 32-week fetal atlas white matter and CSF mask, six head motion parameters, and band-pass filtering at 0.008–0.09 Hz. To create a subnetwork model of the fetal brain, the complete set of unique *n* = 19,306 ROI-pair functional connectivity (FC) (Fisher-z) values for data from all participants were averaged, producing a 197 × 197 connectivity matrix (Fig. 2). The set of averaged correlations across subjects was thresholded and binarized at multiple Fisher-z values, corresponding to edge density sparseness thresholds ranging from 1 to 10% of all possible surviving connections at steps of 0.1%, to generate 91 total adjacency matrices. Connections between ROI pairs separated by <10 mm were removed to minimize the effects of blurring in the spatially normalized fMRI data. The Infomap community detection algorithm [70] was used to assign ROIs to neural subnetworks based on maximization of within-module random walks applied to adjacency matrices at each threshold. Solutions for each threshold were combined using an automated consensus procedure to provide a single model of the community structure by maximizing the normalized mutual information of groups of neighboring solutions and then maximizing modularity [71]. This network solution enabled network-pair-level analyses for the full group (Fig. 2) and reduced the overall number of possible comparisons by more than 150-fold.

### Examination of NAS-related differences in fetal functional connectivity

Exploratory and confirmatory factor analyses were used to generate a single multi-measure factor assessing maternal prenatal negative affect/stress (NAS). Analyses and factor loadings are provided in the Supplemental Material. The resulting NAS score was adjusted for gestational age at scan by computing residual values using the regression NAS–GA + error and used for all subsequent analyses. Enrichment analyses were performed to identify within- or between- network pairs with a significant clustering of strong RSFC-NAS correlations. This enrichment approach has recently been applied to functional neuroimaging studies of fetuses and infants [43, 68, 71, 72], and is an adaptation of methods used in large-scale genome association studies [73–75]. The approach applies a p-threshold of 0.05 to all ROI-pair correlations in the group Fisher-z-transformed FC matrix then uses a Chi-square test to assess the level clustering of strong FC-NAS correlations for within- and between-network ROI pairs. Significance for χ^2^ enrichment is determined by randomly permuting the subject pairing of FC and NAS values 10,000 times [71, 73]. Only networks that were significantly enriched for FC-NAS (χ^2^, df = 1, *p* < 0.05) were treated as significant findings.

### Mixtures-modeling of brain-stress relationships

To explore whether the associations between maternal NAS scores and fetal brain connectivity were buffered by the effects of social support or adaptive health behaviors, we first used exploratory factor analyses to reduce dimensionality across ten measures of social environment and health behavior. Analysis of scree plots with actual and resampled data and Bartlett’s test for sphericity both confirmed that these variables were best summarized as three factors, one linked to health (“Health Engagement”; HE), one to family conflict/cohesion (“Family Systems”; FS) and a third that was a mixture of sleep, medical adherence, substance use, and ECR-R interpersonal avoidance (“Health and Adult Relationship”; HAR) (SI Appendix, Fig. S2). Next, to further reduce dimensionality, we applied a mixtures-based modeling strategy, weighted quantile sum (WQS) regression [76, 77], to construct a single empirically-estimated connectivity index (i.e., WQS stress-connectivity index), summarizing the overall magnitude of RSFC effects in a given fetal brain. In a third step, initial univariate models were constructed to evaluate relationships between the WQS stress-connectivity index and maternal NAS scores without additional health and social support factors. Finally, the models were rerun adding the health and social support factors to examine both effect modification and to test for interaction effects. In all models, a robust cross-validation strategy was applied, such that the data were divided into training (40% of data) and validation (60%) sets, with model weights estimated in the training set before application to the validation set, both to avoid over-fitting and ensure the generalizability of results. Additional information about WQS model estimation is provided in Supplemental Material.

### Evaluation of prenatal NAS, fetal brain FC, and gestational age at delivery

We evaluated relationships between maternal prenatal NAS, fetal brain, and age at delivery. Standard linear models were used to evaluate these relationships, using either maternal stress, individual between-network connectivity values, or the stress-connectivity WQS mixture, derived as above-described, as predictors.

## Results

### Fetal brain targets of maternal NAS

**Fig. 3:**
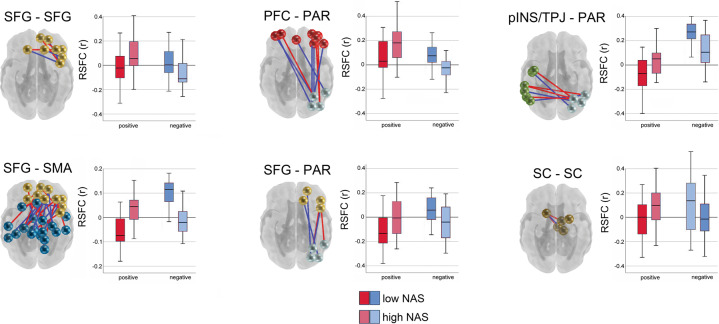
RSFC across and within large-scale fetal brain networks is bidirectionally linked to variation in a factor representing cumulative maternal prenatal negative affect and stress (NASF). Connections that are stronger in fetuses of mothers with higher NASF are plotted in red, and the reverse is plotted in blue. Boxplots denote global mean centered r-values for the top and bottom NASF quartiles, averaged across positive or negative network edges within each network pair. SFG superior frontal gyrus, SMA somatomotor network, SC subcortical gray matter, pINS/TPJ posterior insula/ temporoparietal junction, PAR parietal, PFC prefrontal cortex.

Community detection analysis generated a 16 functional network consensus model (Fig. 2) that became the basis for isolating significant relationships between maternal prenatal NAS and between and within network RSFC. Results of enrichment analysis demonstrated that effects of maternal prenatal NAS were evident in six fetal network pairings. Specifically, maternal prenatal NAS factor scores were related to variation in FC values between the (i) superior frontal and sensorimotor networks (SFG–SMA), (ii) a left posterior insula/temporoparietal junction and a right superior parietal network (pINS/TPJ–PAR), (iii) superior frontal and parietal networks (SFG–PAR), and (iv) prefrontal and parietal networks (PFC–PAR). In addition, maternal prenatal NAS scores were related to FC within the (v) subcortical striatal network (SC–SC), and the (vi) superior frontal gyrus network (SFG–SFG). Significant between and within network effects were confirmed with χ^2^ tests for independence. A summary of connections comprising significant enrichment results is provided in Table 1 and between-network positive and negative significant connections are visually represented in Fig. 3.

**Table 1:** Enriched networks based on relationship between NAS and fetal RSFC.

Network pair	WQS Weight	Enrichment c^2^	Enrichment p-val
SFG–SMA	0.43	8.55	0.01
SC–SC	0.29	4.66	0.05
pINS/TPJ–PAR	0.13	10.73	0.004
SFG–PAR	0.08	4.59	0.05
SFG–SFG	0.04	10.05	0.007
PFC–PAR	0.04	4.88	0.05

The number of significant connections within each network pair varies from region to region. Enrichment c^2^ statistics and *p*-values describe the degree to which the number of significant ROI–ROI pairs within each network pair was greater than could be expected by chance.

### Moderation of effects by social support and health behaviors

We further evaluated relationships between fetal connectivity and maternal stress in a mixtures-based strategy. A weighted quantile sum (WQS) model replicated findings from enrichment and χ^2^ tests, showing that NAS and connectivity across the 6 networks was significant as a mixture (β = 0.82, *p* < 0.001) and highlighting that the strongest associations were in connectivity of SFG–SMA, pINS/TPJ-PC, and SFG–PAR network pairs (SI Appendix, Fig. S3). Next, we tested for moderation by examining potential interactions in WQS models that included health and social support factors. We found no significant associations between NAS and factors corresponding to diet, exercise (factor *HE*), and family conflict/cohesion (factor *FS*), but found that a factor comprising sleep, medical adherence, substance avoidance, and low interpersonal avoidance (factor *HAR*), significantly moderated (β = 0.16, *p* = 0.02) the relationship between the maternal prenatal NAS and the WQS stress-connectivity index. The effect was such that participants with lower HAR scores had weaker associations with the stress-connectivity index, but higher stress overall. In the context of this interaction, connectivity between PFC–PAR and SFG–SMA networks were most significantly related to HAR (*p* < 0.000; SI Appendix, Table S4).

### Maternal NAS-related brain FC relates to gestational age at delivery

**Fig. 4:**
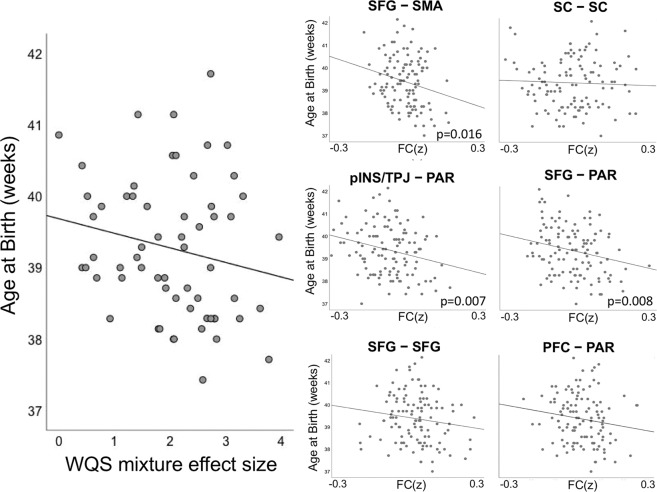
Effects of maternal stress in the fetal brain relate to gestational age at delivery. The left panel depicts the negative association (β = −0.18, *p* = 0.05) between the WQS stress-connectivity index and gestational age at delivery. Thus, stress-related changes in fetal connectivity are significantly associated with shorter gestational periods. The right panels depict average connectivity across network edges significantly associated with maternal stress for each subject for each of the six significant network pairs (FC(z)). Significant associations between strength of stress-connectivity effect and gestational age at delivery was observed in the SFG–PAR (β = −2.46, *p* = 0.008), pINS/TPJ–PAR (β = −2.68, *p* = 0.007), and SFG–SMA (β = −3.52, *p* = 0.016) network pairs.

Maternal NAS scores were associated with younger gestational age at delivery (β = −0.36, *p* = 0.003), in agreement with prior literature [48, 50]. Here, we extended our analysis of stress-connectivity indices to consider potential associations between stress correlates in the fetal brain and fetal gestational age at birth. Our findings indicated a marginally significant negative association (β = −0.18, *p* = 0.05) between the omnibus WQS stress-connectivity index and gestational age at delivery. That is, a single measure reflecting the effect of stress on neural connectivity in each fetus was associated with how early that fetus was born (see Fig. 4). Given the marginal significance of this relationship, we pursued follow-up analyses with traditional linear models to investigate the association between NAS and length of gestation in each network pair. Applying Holm–Bonferroni correction to six tests of connectivity to gestational age at birth, we observed significant associations between gestational age at delivery and connectivity between SFG–PAR (β = −2.46, *p* = 0.008), pINS/TPJ–PAR (β = −2.68, *p* = 0.007), and SFG–SMA (β = −3.52, *p* = 0.016) network pairs (Table 2).

**Table 2:** Regression of NAS and RSFC on infant birth age.

Predictor	β	SE	B	*p*-value
NAS	−0.36	0.12	−3.04	0.003
PFC–PAR	−1.90	1.15	−1.66	0.099
SFG–SFG	−1.67	1.07	−1.56	0.120
SFG–SMA	−3.52	1.44	−2.45	0.016
SFG–PAR	−2.46	0.92	−2.68	0.008
pINS/TPJ–PAR	−2.68	0.97	−2.76	0.007
SC–SC	−0.39	0.75	−0.52	0.607

Regression of stress-related RSFC and age at delivery are provided for each network. Regression was performed in the full sample of 118.

### Evaluation of potential confounds

Tests of associations between maternal demographics, data quality control (QC) parameters, and maternal NAS residuals used in the enrichment analysis were largely not significant, suggesting that several factors with potential to confound observed stress-brain mixtures do not appear to have influenced analyses performed in this dataset. Maternal NAS residual scores were not related to maternal age (r = 0.018, *p* = .85), gross income (r = −0.061, *p* = 0.55), average translational motion (r = −0.109, *p* = 0.24), or frame count (r = 0.123, *p* = 0.18). However, maternal NAS residual scores were significantly related to average rotational motion (r = −0.185, *p* = 0.045). Consequently, we performed additional analyses testing the relationship between motion and significant between-network connectivity effects across subjects. Connectivity values were not related to motion for the majority of network pairs, but a correlation was observed between rotational motion and SFG–SMA connectivity (r = −0.24, *p* = 0.009). This correlation does not surpass Bonferroni correction for multiple tests, but is nonetheless highlighted and reported in Appendix, Table S5. As an additional step, we confirmed that motion measures and the overall WQS connectivity index were not significantly related, WQS and XYZ motion, r = 0.008, *p* = .951, and WQS and PYR motion, r = −0.165, *p* = .203. Additional contextual variables of interest in our analyses, specifically social support and health behaviors, did have the expected relationship with maternal NAS: NAS scores were negatively correlated with prenatal ECR-R avoidance (r = −0.46, *p* < 0.001), diet (r = −0.23, *p* = 0.016), medical adherence (r = −0.29, *p* = 0.001), and sleep (r = −0.34, *p* < 0.001), and positively associated with substance abuse (r = 0.41, *p* < 0.001). Maternal NAS was not related to other social support and health behaviors subscales: ECR-R anxiety, FES-R cohesion, FES-R expressiveness, FES-R conflict, exercise.

## Discussion

From a developmental origins of health and disease (DOHaD) perspective, this research provides a framework to evaluate the very early embedding of biological risk in social and behavioral contexts [78, 79]. Research in neonates and young children has suggested that prenatal stress hormones act on various signaling receptors that interact with genetic/epigenetic factors and additional environmental input to influence brain development prior to birth [80]. Animal models of early life stress have bolstered this view, providing evidence that mouse pups stressed in the first weeks of life (equivalent to human third trimester) show shorter and less complex dendrites, hypomyelination, and altered synaptogenesis [23–26]. However, whether or not neurodevelopmental trajectories begin to be altered in the womb has remained a long-standing question in the field of human prenatal stress programming. This study provides evidence that maternal prenatal stress and negative affect are associated with alterations in human brain networks before birth. We report differences in fetal system-level dynamics (i.e., functional connectivity) thus extending models of biological embedding of stress by propelling us back to the most delicate time in human brain maturation, where change is more rapid than at any other time in the life course.

Alterations in fetal functional connectivity were apparent between superior frontal and motor regions and in cross-hemispheric connectivity of posterior insula and temporoparietal brain regions (pINS/TPJ–PAR). These observations are of interest given that connectivity between these regions increases with advancing fetal age [40, 81], and abnormal connectivity in these regions has been reported among fetuses and neonates born preterm [45, 82, 83]. Such studies confirm the importance of connectional processes taking place between these regions during the fetal period and lead to suggestion that changes to these processes may have long-ranging effects. Interestingly, temporo-insular regions have also been implicated in the generation of spontaneous functional activity during late stages of fetal development [46, 47], leading to suggestions that these spontaneous functional bursts of activity may be foundational to establishing the organizational properties of the human functional connectome architecture [46].

We also observed significant differences in RSFC between anterior and posterior frontal and parietal regions (SFG–PAR; PFC–PAR) and connectivity within the superior frontal gyrus (SFG–SFG) in fetuses exposed to greater stress in utero. Research in neonates and infants confirms that these networks are evident early in human life [84], and research in adults suggests a potential transdiagnostic role of disrupted frontoparietal network connectivity in human affective, psychiatric, and neurological disease [85]. Further, alterations in frontoparietal networks have been shown to be experientially dependent, as evidenced in longitudinal studies of acute psychosocial stress exposure [86] and cross-sectional investigations of RSFC among individuals with childhood trauma exposure [87]. We also observed right laterality in our stress-related frontoparietal RSFC differences. While few, if any, studies of emotional psychopathology have specifically addressed laterality of frontoparietal RSFC disruptions, the important role of the right hemisphere in processing emotions is widely studied [88]. Further, animal models of induced depression have reported laterality in differential gene expression in frontoparietal regions, specifically, right lateralized effects in animals that show reduced resilience to stress exposure [89]. Convergence of our human fetal results with these known priors suggests that further examination of both early childhood onset of affect-related frontoparietal disturbance, including examinations of laterality, are warranted.

In addition, we observed prenatal maternal stress-related differences in RSFC within a fetal subnetwork encompassing areas of the striatum. The effect of stress on the striatum has been shown to relate to neuronal microstructure [90], gene and neurochemical expression [91], and connectivity [92–94]. Studies both in humans and in animals confirm that restructuring of the striatum following stress has behavioral relevance, most strongly impacting domains of social behavior, decision-making, affective valuation, and risk for internalizing illness [90–93]. It is possible that prenatal variation in striatal subnetwork functional connectivity predisposes individuals to long-term processing differences across these and other related behavioral domains. Our study provides evidence to support this claim, and suggests that combined changes in the striatum, insulo-temporal and frontoparietal brain systems may underlie long-term stress-related behavioral effects. It is noteworthy that across network pairs the observed relationships between maternal NAS and fetal functional connectivity were comprised of a mix of both positive and negative associations, without clear directionality. That is, both augmented and diminished network connectivity was observed. This finding is not unexpected when studying subnetworks comprised of numerous nodes that are separated in space and/or expansive. This approach has the advantage of enabling examination of whole-brain connectivity, which is particularly favorable in the fetal brain where priors are presently rather limited. However, a natural next step will be to address directionality within specific circuits and at finer resolution.

Individual responses to stress vary widely. A question we sought to address after confirming the existence of prenatal alterations in human brain RSFC was whether specific protective variables explained variation in maternal NAS-related brain differences. The unique sample recruited for this study was predominately low-income, unpartnered, minority women drawn from a community with elevated levels of stress and violence. This is an important population to work with both because this is a population with considerable need, and because meta-analysis indicates associations between maternal prenatal stress and child outcomes are strongest in high-risk groups [95]. It is known that there are specific traits, such as internal locus of control, social skills, exercise, maternal mindfulness, optimism, and ego development that predict more resilient outcomes [96–99]. For example, Christianson and colleagues showed that exercise mitigates the expression of stressor-induced anxiety; [100] Young and colleagues showed that responses to stress are attenuated by strong male bonds in wild macaques; [101] and Wellman and colleagues showed individual differences in the effects of stress on REM in Wistar rats and suggest that sleep may be an important biomarker of stress resilience and vulnerability [102]. We found that a latent factor comprising social support and health, specifically, sleep, medical adherence, avoiding alcohol and cigarettes, and romantic closeness, was a significant modifier of the association between maternal stress and fetal functional connectivity (SI Appendix, Fig. S4), such that stress had reduced impact in fetuses of less healthy mothers. Although seemingly counterintuitive, these findings are consistent with evidence that attenuated stress, physiological, and immune responses occur under conditions of chronic stress [103, 104]. That is, when prenatal conditions are more challenging, it may adaptive for the fetus to be less sensitive to potential modifiers, such as stress. In contrast, under less challenging prenatal conditions, stress may have potential to have greater effect. From a public health standpoint, the main effects of NAS on the fetal brain continue to point to specific interventional targets for reducing the effects of prenatal stress programming, including sleep and relationship support, ease of access to medical care, and resources targeting reduced substance use. One caveat of the current study is narrow focus on high-risk women; it will be important for future research to evaluate replication of these observations in samples with varied sociodemographic composition.

Finally, we discovered that a greater overall magnitude in the association between maternal NAS and fetal functional brain alteration was associated with a shorter gestational course. Prior studies have reported a negative association between maternal stress and fetal age at delivery, but to our knowledge this is the first study to report that the magnitude of response to stress in the fetal brain may additionally explain variability in birth outcomes. Importantly, these data alone do not suggest that changes in the fetal brain mediate early delivery. Instead, the relative magnitude of the association between maternal prenatal stress on fetal neurobiology is likely reflective of unobserved factors that impact delivery timing, including broader stress-related alterations in perinatal biology and physiology. Here, these associations were observed in the 3 weeks prior to delivery. It will be important for future work to test whether interactions between stress, timing of delivery and fetal brain FC extends to fetuses subsequently born preterm.

In research that considers offspring correlates of maternal stress, negative outcomes are frequently emphasized. However, in the original Barker hypothesis [105] in utero adaptations are oriented around promoting fetal/offspring survival. Within that context, fetal brain differences reported here may reflect adaptations of the fetus to best meet the challenges of the harsh environment contributing to maternal stress. The present study does not include assessment of child outcomes, which limits ability to address the relevance of fetal brain adaptations to future child well-being. Furthermore, maternal stress is not easily decoupled from confounding contextual and health factors. As an example, we did not gather information about illicit and prescription drug use and are thus unable to evaluate potential contribution of those factors to observed effects. An additional consideration in studies that examine offspring correlates of maternal prenatal stress is that mother and fetus share genetic liability that is important in the patterning of neural circuitry. Overall, in studies such as this, attribution about causality and directionality are not warranted, and much remains to be done to understand how the specific intrauterine signaling factors promote or hinder optimal welfare of the future child.

Overall, our data suggest that widespread differences in the fetal brain are related to maternal self-reported stress and negative affect during pregnancy. The findings advance prior human studies that have demonstrated associations between prospective measures of psychological and biological indices of stress and brain structure and function [27–31, 106]. Empirical evidence presented here, along with data presented in those influential prior studies, support the notion that excess stress in utero has the potential to affect neural development with implications for future health across the lifespan.

## Supplementary information

Supplemental Material
