# Supplementary material for: Interactive relations between maternal prenatal stress, fetal brain connectivity, and gestational age at delivery
Source: Neuropsychopharmacology. 2021 Jun 29;46(10):1839–47. doi: 10.1038/s41386-021-01066-7 (PMC8357800; doi:10.1038/s41386-021-01066-7)
Supplement: Supplementary file 1 — Supplemental Material [file 41386_2021_1066_MOESM1_ESM.docx]

*Supplemental Material*

*Title:* **Maternal prenatal stress and environment: effects on fetal brain and timing of delivery**

*Authors:* Moriah E. Thomason, Jasmine L. Hect, Rebecca Waller, Paul Curtin

**Participant Selection**. A total of 221 women were enrolled during pregnancy between 25 and 39 weeks of gestation. Only pregnant women between 18-40 years old, native English speaking, singleton pregnancies, and cases with normal fetal brain anatomy as assessed by ultrasound and MRI examination were eligible to participate. Exclusions were assessed based on availability of preprocessed imaging data and/or presence of birth complications. Specifically, cases were excluded if fewer than 100 low movement functional MRI timeframes were available, and/or if the fetus was subsequently born prior to 37 weeks or with birth weight <2500 grams. A summary of case exclusions is provided in *SI Appendix,* *Table S2*. The selected study sample did not differ from the non-study population in race/ethnicity, child sex, health behavior, maternal age or demographic factors *SI Appendix,* *Table S6*.

**MRI Scanning.** MRI data were acquired on a Siemens Verio 70-cm open-bore 3T MR system using a 550 g abdominal 4-Channel Siemens Flex Coil (Siemens, Munich, Germany). Resting-state fMRI data were acquired using a gradient echo planar imaging sequence: TR/TE 2000/30 ms, flip angle 80°, 360 frames, axial 4 mm slice thickness, voxel size 3.4×3.4×4 mm^3^), repeated twice. Between 12 to 24 minutes of fetal resting-state fMRI data were collected per participant, average estimated specific absorption rate (SAR) was 0.20 W/kg, SD = 0.07.

**Correlations between 5 NAS subscales and connectivity between each significant network pair.** In a *post hoc* analysis, each of the 5 scales comprising the NAS score (i.e. CES-D, STAI, PSWQ, PSST, and SWLS) were tested separately for strength of their association with average connectivity for each network pair: PFC-PAR, SFG-SFG, SFG-SMA, SFG-PAR, pINS/TPJ-PAR, and SC-SC. This was done to address whether results would differ if we had used individual scales rather than a summary NAS score as our predictor. Using inverse r-values for ROI-ROI pairs that were negatively correlated, we computed the average r-value for each network pair and tested associations using two-tailed Pearson’s correlations that are reported in *SI Appendix,* *Table S7*. This analysis confirmed that individual factors represented in the NAS score were each associated with fetal FC in the expected directions.

**Maternal psychological versus physiological stress: subsample examination of maternal prenatal salivary cortisol concentration.** It is well known that over the course of pregnancy, circulating maternal cortisol levels dramatically rise, due in part to both increased corticotrophin-releasing hormone (CRH) secretion from the placenta and increased estrogen, which results in more bioavailable cortisol due to increased activation of corticosteroid-binding globulin. In later stages of pregnancy increased circulating cortisol leads to maternal thalamic CRH downregulation. This results in an attenuation of physiological and psychological stress reactivity in later stages of pregnancy.^7-9^ Here, in a subset of cases (N = 54) with salivary cortisol measured at the time of MRI, we sought to replicate the finding that maternal cortisol levels increase over pregnancy, and in an exploratory analysis, test associations between cortisol and psychological measures of negative affect and stress. Pearson correlation confirmed a significant increase in cortisol with week of pregnancy, r = .57, p < 0.001. However, tests of the associations between cortisol and the 5 self-report measures that went into the cumulative NAS score and the NAS score, itself were not significant. Specifically, we observed: PSST r = .05, p = .75; SWLS r = -.09, p = .54; STAI r = .06, p = .65; PSWQ r = .22, p = .12; CESD r = .04, p = .76; and NAS r = .13, p = .36. It is possible that this small sample size rendered us underpowered to detect significant associations, and that individual differences not factored into these models (e.g., exercise, novelty, time of day, substance use ^10-13^ may be contributing to variation in effects. An additional complexity is that both heightened and blunted cortisol levels are reportedly associated with mood disorders, trauma exposure, and poor health.^14-16^ Curvilinear relationships between cortisol concentration and perceived stress have also been reported, such that cortisol concentrations increase with higher perceived stress but decrease at the highest level of stress.^17^ Overall, while cortisol was not planned as a primary measure of prenatal maternal stress, there remains opportunity for future research to address the role of cortisol in prenatal brain development in a larger sample, and using more stable cortisol parkers such as hair samples and diurnal measures.

**Weighted Quantile Sum (WQS) Mixtures-modeling of brain-stress relationships.** WQS regression is a relatively recently-developed technique for supervised dimensionality-reduction that has become increasingly common in recent years, particularly in the context of “mixtures analysis” which examine combinatorial effects.^20,21^ In neuroscience studies, this approach has primarily been applied in the context of integrating high-dimensional chemical and social exposures data, and linking to cognitive, behavioral, and structural and functional neuroimaging data.^22-26^ In the present study, WQS was used to generate a subject-specific index corresponding to the empirically-estimated overall effect of stress on fetal neural connectivity and to confirm significance of neural effects in an omnibus mixture model. The WQS ensemble method enables supervised dimensionality-reduction, whereby a set of predictors – here, functional connectivity values for connections across 6 brain network pairs – are used to construct a single empirically-estimated connectivity index (i.e., WQS stress-connectivity index), which is then evaluated in a standard linear model. Prior to WQS model estimation, negative connectivity values were transformed by taking absolute values (i.e., ABS(x)). This reversal of negative values was necessary so that positive and negative edges would not cancel one another out in estimation of magnitude of effects within a network pair. The WQS stress-connectivity index was calculated such that $WQS=\sum w_{i}q_{ij}$, where *w_i_* indicated a vector of estimated weights estimated for each brain region based on its association with maternal NAS scores, and *q_i,j_* indicates the ranked (deciled) connectivity measure for each subject and region. Weights were estimated across 100 bootstrapped samples, with weights constrained to vary between 0-1 and to sum to 1. Use of a standard linear model, as $g\left( \mu\right)=\beta_{0}+ \beta_{1}WQS+z'\varphi$, where $g\left( \mu\right)$ reflected an identity-link to maternal NAS scores, $\beta_{0}$was the model intercept, and $\beta_{1}WQS+z'\varphi$ indicated a regression parameter associated with the WQS stress-connectivity index and associated covariates and parameters ($z'\varphi$), allowed us to directly test the association between the mixture of connectivity values and maternal NAS scores. This model thus simultaneously allows the evaluation of the combined effect of multiple brain regions, while also allowing for the dissection of discrete effects associated with individual regions, which are apparent in the magnitude of associated weights.

An additional benefit of this approach is in providing a generalizable linear framework for hypothesis testing. Critically, this allows us to leverage the flexibility of the linear modeling framework to adjust our effect estimates for key biological covariates, as well as extend our analysis to include multiplicative effects, particularly the interaction with stress-based measurements derived from factor analysis. While other comparable multivariate methods such as principle component analysis, linear discriminant analysis, partial least squares, and related approaches might provide for dimensionality reduction, they do not similarly allow for covariate adjustment and multiplicative effects testing in a comparable manner with direct hypothesis-based (p-value) testing. Further, with respect to the specific nature of neuroimaging data, WQS also offers advantages in that the approach was developed specifically to address the challenge of estimating effects in contexts of high-dimensional and highly correlated data. This is achieved through 1) the imposition of constraints during the estimation of effects; and, 2) the use of ensemble methods – here, bootstrapping – during effect estimation, which simulation studies demonstrate can be highly effective in ameliorating misestimation by collinearity even in contexts where correlations exceed r>0.9, and the number of predictors exceeds the number of samples. ^21^

**Combination of positive and negative values to estimate overall effect for network pairs.** We performed an additional analysis to address whether taking the average of inversely transformed (i.e., ABS(x)) negative and positive FC values was substantially different than discarding all negative values and taking the average of only positive FC values. After computing individual participant magnitude of stress effect using each of these approaches, we applied Pearson correlation analysis for each significant network pair. We found that values derived for each subject in each of these ways were highly correlated across the 6 networks tested (*r*-values range = 0.6903 to 0.8442), suggesting that WQS would not yield a different result if this alternate approach had been used.

**Classification of Social Support and Health Behaviors.** To explore whether social support and adaptive health behaviors buffered against associations between NAS scores and fetal brain development, we used a data reduction technique to generate multi-construct factors across measures assessing either social support or health behaviors. First, social support was measured using two measures: the Family Environment Scale FES-R; ^24^ a 27-item measure that assess the respondent’s current perception of underlying dimensions of the family environment, comprised of 3 subscales: cohesion, expressiveness, and conflict; and the Experiences with Close Relationships Scale ECR-R; ^25^ a 36-item measure of adult relationship style, comprised of 2 subscales: avoidance and anxiety. In general, based on ECR-R scores, avoidant individuals find discomfort with intimacy and seek independence, whereas anxious individuals tend to fear rejection and abandonment. Second, health behaviors were measured using an adapted version of the Health Practices Scale HPS; ^26^ a 53-item measure comprised of 5 subscales: diet, exercise, medical adherence, substance abuse, and sleep. Items were rated on a 6-point Likert Scale ranging from (1) Never to (6) Always. Psychometric evaluation of the adapted health measure is provided below. To generate multi-construct factors assessing the intersection of social support and adaptive health behaviors, raw scores for each subscale from across the FES-R, ECR-R, and HPS (i.e., 10 in total), were transformed such that higher scores corresponded to positive behaviors for use in exploratory factor analysis (EFA), implemented in *R* v3.5.2 with the *psych*, *nFactors*, and *GPArotation* packages. Factors were extracted using maximum likelihoods with an oblique (oblimin) rotation.

**Psychometric Evaluation** **of Adapted Health Measure.** Based on prior work demonstrating that health questionnaires can be sensitive, stigmatizing, less reliable, or inaccessible in high-risk, low SES and/or racial/ethnic minority individuals, ^27-29^ thirteen items on the HPS questionnaire were removed or revised. Description of HPS modifications are provided below. We followed three steps to test the psychometric properties of the newly-adapted version of the health practices scale HPS; ^26^ measure: (i) Compared alpha coefficients of new subscales to those of the original subscales from Jackson (2006) to have confidence in the reliability of new subscales; (ii) Compared magnitude of inter-subscale correlations based on new subscales to those reported in Jackson (2006); (iii) Performed confirmatory factor analysis (*not* done by Jackson, 2006) to obtain model fit statistics for a five factor model (i.e., items loading onto separate diet, substance use, medical, exercise, and sleep factors as proposed by Jackson (2006). Summary of original and newly added health behavior items are provided in *Supplemental Table S8*. We find that: (i) Alpha coefficients of new subscales indicate acceptable-to-good internal consistency comparable with the estimates reported by Jackson (2006), except for Medical (lower for our sample): Diet (17 items), α = .86; Exercise (8 items), α = .82; Medical (6 items), α = .67; Sleep (5 items), α = .84; Substance use (9 items), α = .77; (ii) The inter-subscale correlations were moderate, consistent with the estimates reported by Jackson (*Supplemental Table S9*); (iii) Confirmatory Factor Analysis in Mplus vs. 7.2 using Weighted Least Squares Means and Variance (WLSMV) adjusted estimation, appropriate for ordinal scales, indicated that the items loaded onto a five-factor model consistent with hypothesized factors (CFI=.92, TLI=.91, RMESA=.05, SRMR=.096; *Supplemental Table S10*). Readers may note that based on item loadings it may be advisable to remove the following questions in future studies: (i) Go to the same dentist office for all my dental care, (ii) Eat fast food, (iii) Pay attention to the number of calories in the foods I eat, and (iv) Read food labels to see what I am eating. These have overlapping item content with other questions, and when the model is re-run after removing these items, the fit remains good.

**Future Directions.** Primary considerations for the present study are that fetal fMRI is a young field and with many challenges in acquisition and analysis remaining to be addressed, and that much remains to be done to understand how maternal stress biology programs patterning of the fetal brain. Notably, empirically-derived, innovative solutions in both fetal data acquisition ^31,32^ and processing ^33-36^ are emerging. Further examination of fetal fMRI methodology is available in recent reviews ^37-39^ and schematized in select empirical papers.^33,40-42^ An important direction for future studies will be to extend analyses to other imaging modalities, such as anatomical, diffusion, and spectroscopy imaging, to understand physical and chemical differences that may accompany variation in fetal functional circuitry. Indeed, there are a number of biological processes and pathways through which maternal stress biology acts to influence fetal neurological development. At the forefront of these are the maternal-placental-fetal endocrine signaling system and inflammatory pathways. The instructive role that these systems play on neuronal and glial cell proliferation and migration, synapse formation, myelination, and other essential growth processes has been the subject of several influential prior reviews.^43-45^ Heightened exposure to endocrine and inflammatory signals, especially interleukin-6 and tumor necrosis factor–α, alters bioavailability of key neurotrophic and growth factors, neurotransmitters, and thyroid hormones. A recent review by Moog and colleagues describes the role of thyroid hormones in brain growth and development and highlights that even more moderate forms of maternal thyroid dysfunction, particularly during early gestation, may have a long-lasting influence.^46^ An important direction for the advancement of clinical science will be to isolate specific actionable biochemical pathways that could serve as targets for novel intervention strategies in the future.

**Data and code availability**

The corresponding author makes fetal fMRI datasets available via the NIMH/NIH NDAR database (<https://ndar.nih.gov/edit_collection.html?id=2434>) and OpenNeuro (<https://openneuro.org/datasets/ds003090>), and shares processing pipelines (<https://github.com/saigerutherford/fetal-code>) and templates ([www.brainnexus.com](http://www.brainnexus.com)). Access to additional data can be arranged via direct request to corresponding author MET.

**SUPPLEMENTAL FIGURES**


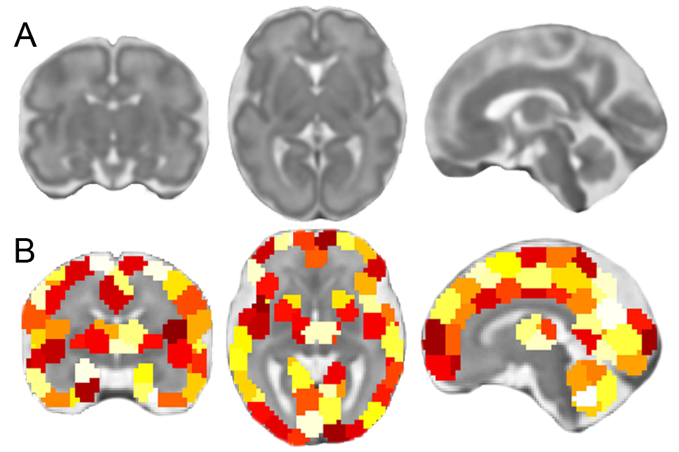


**Supplemental Figure 1.** Pycluster parcellation. All fetal functional data was normalized to a 32-week fetal brain template (A) and parcellated using Pycluster to generate 197 spatially contiguous, similarly sized ROIs across the cortex, subcortex, and cerebellum (B). The resulting parcellation file is available via the NIMH/NIH NDAR database (<https://ndar.nih.gov/edit_collection.html?id=2434>).

**
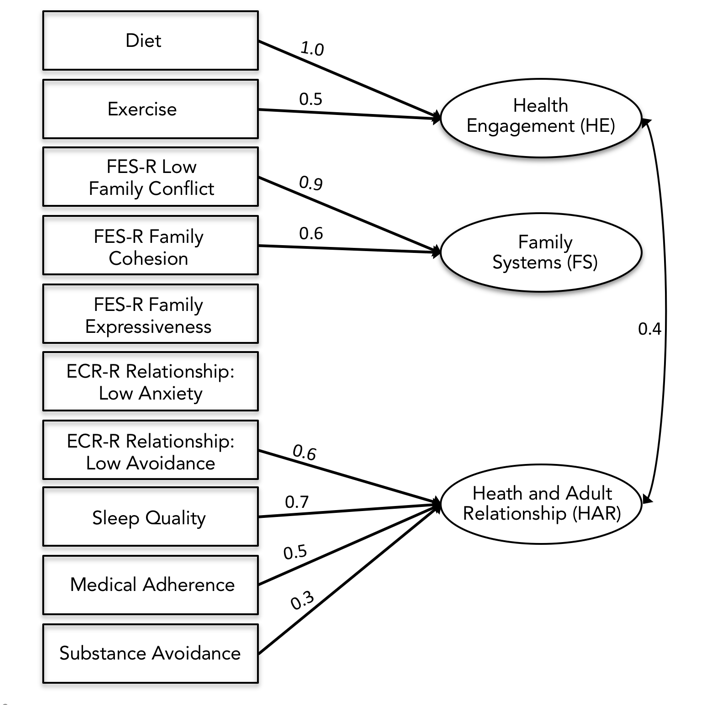
**

**Supplemental Figure 2.** Factor structure underlying social support and adaptive health behaviors. Circles denote Health Engagement (HE), Family Systems (FS), and Health and Adult Relationship (HAR) factors extracted from FES-R. ECR-R, and HPS scales. Lines indicate correlations among factors, or correlations (loadings) between raw scores (boxes) and corresponding factors. Numbers indicate correlation coefficients.

**
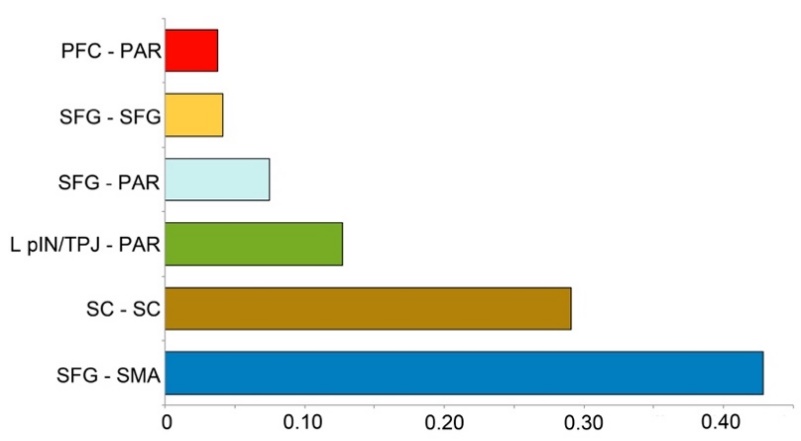
**

**Supplemental Figure 3.** WQS weight describes the contribution of each significant network pair onto the WQS stress-connectivity index. The WQS model replicated findings from enrichment and χ^2^ tests, showing that NAS and connectivity across the 6 networks was significant as a mixture (β=0.82, p <0.001) and highlighting that the strongest associations were in connectivity of SFG-SMA, pINS/TPJ-PC, and SFG-PAR network pairs.


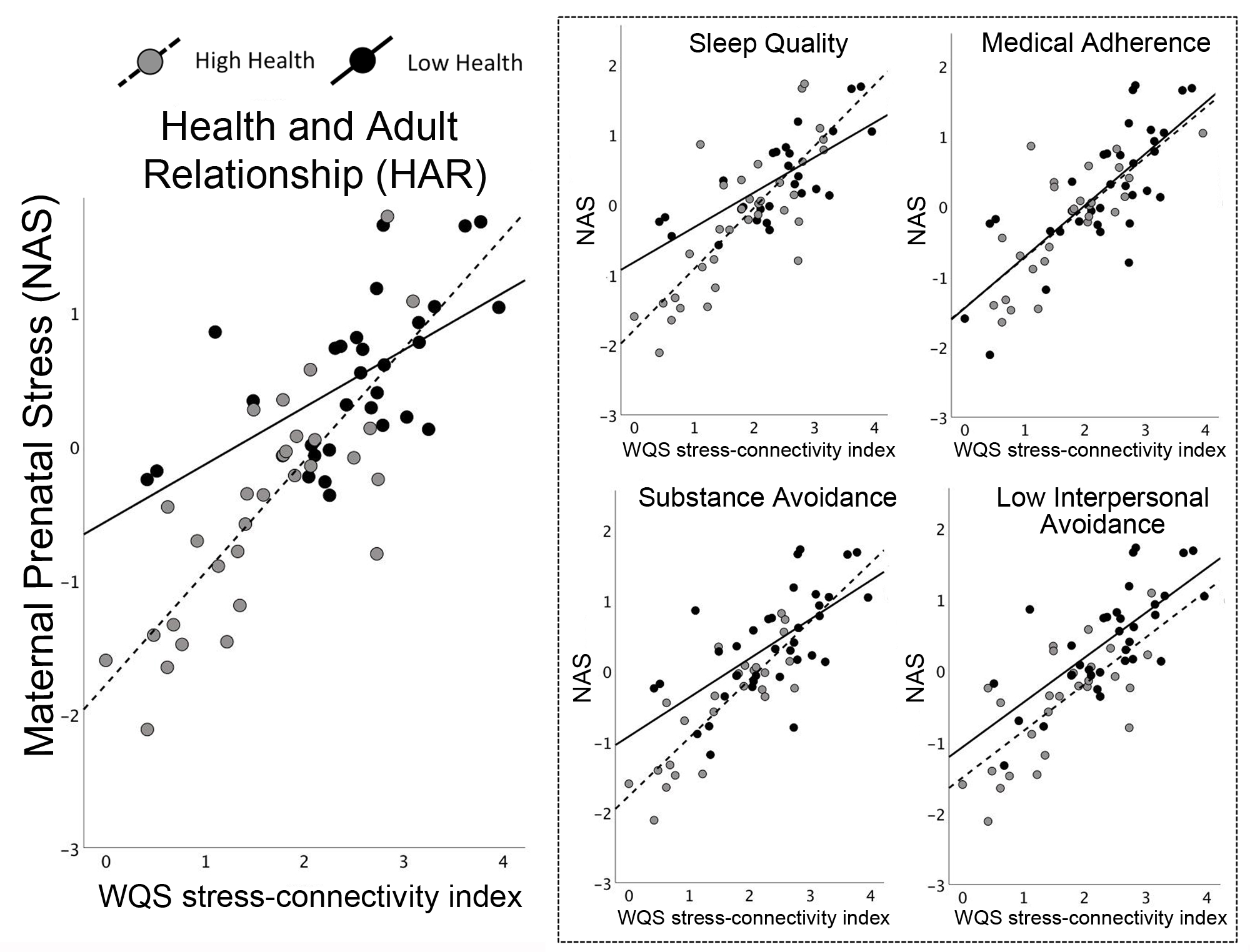


**Supplemental Figure 4. Maternal health factor and stress-brain mixture relationship by sub-scale.** To describe the nature of the interaction of Health/Adult Relationship (HAR), prenatal stress, and the weighted quantile sum (WQS) stress-connectivity index, HAR was dichotomized by median split for better visualization (left). The subscales of HAR, sleep quality, medical adherence, substance avoidance, and relationship involvement, are also plotted against NAS and WQS (right) and demonstrate a unique contribution of sleep quality and substance use on the stress-brain relationship to this interaction, in comparison to affiliative relationship and medical adherence.

**SUPPLEMENTAL TABLES**

**Supplemental Table 1.** Sociodemographic characteristics and birth outcomes (N = 118)

| **Outcome** | **Value** |
| --- | --- |
| Maternal age, years, M (SD) | 25.1 (4.5) |
| Race/ethnicity, n (%) |  |
| African American | 97 (82.2) |
| Caucasian | 11 (9.3) |
| Bi-racial | 4 (3.4) |
| Not disclosed | 6 (5.1) |
| Relationship status, n (%) |  |
| Single | 69 (58.5) |
| Married/Partnered | 41 (34.7) |
| Divorced | 1 (0.8) |
| Not disclosed | 7 (5.9) |
| Annual household income, n (%) |  |
| <$10,000 | 42 (35.6) |
| $10,000-20,000 | 23 (19.5) |
| $20,000-30,000 | 19 (16.1) |
| >$30,000 | 16 (13.3) |
| Not disclosed | 18 (15.3) |
| Maternal education, n (%) |  |
| No diploma/No GED | 18 (15.3) |
| GED/high school diploma | 46 (39.0) |
| Some college | 43 (36.4) |
| >=2yr college degree | 4 (3.3) |
| Not disclosed | 5 (4.2) |
| Parity (primiparous), n (%) | 25 (21.2) |
| Child sex, n (%) |  |
| Female | 48 (41) |
| Male | 70 (59) |
| Child characteristics, M (SD) |  |
| Gestational age at scan, weeks | 32.9 (3.9) |
| Gestational age at birth, weeks | 39.4 (1.1) |
| Birth weight, grams | 3338.9 (441.7) |

**Supplemental Table 2.** Summary of case exclusions from current analyses

|  | Frequency | Percent |
| --- | --- | --- |
| Health Considerations* | 39 | 17.6% |
| Below quality control standards | 42 | 19.0% |
| Age at scan <25 weeks | 6 | 2.7% |
| Differential fMRI acquisition | 8 | 3.6% |
| Not processed | 9 | 3.6% |
| Included in NAS analyses | 118 | 53.4% |
| Total | 221 | 100 |
| *Health considerations included (i) infection, n=2, 5%; (ii) born <37 weeks, n=28, 72%; (iii) born <2500g, n=7, 18%; and (iv) congenital abnormality, n=2, 5%. Quality control standards included high motion, n=22, 52%; image artifacts, n=15, 36%; or both, n=5, 12%. | | |

**Supplemental Table 3.** Group descriptive variables for prenatal stress measures

| **Stress Construct** | **Scale Range** | **Respondent Mean (SD)** | **Scale Interpretation** |
| --- | --- | --- | --- |
| Perceived Stress | 0 - 36 | 14.61 (6.41) | >13.7 = above mean for females in USA |
| Life Satisfaction | 5 - 35 | 23.75 (6.55) | <14 = extremely dissatisfied; >19 = not extremely dissatisfied |
| Anxiety | 6 - 24 | 9.96 (2.56) | >15 = high anxiety |
| Worry | 16 - 80 | 42.57 (11.74) | 60-80 = high worry |
| Depression | 8 - 42 | 21.45 (7.60) | >16 = at risk for clinical depression |

**Supplemental Table 4.** Associations between maternal health/socioemotional support measures, NAS, and RSFC of significant network pairs

| **Outcome** | **Predictor** | **Estimate** | **Std. Error** | **p-value** |
| --- | --- | --- | --- | --- |
| **HE** |  |  |  |  |
|  | PFC - PAR | -0.01 | 0.01 | 0.377 |
|  | SFG -SFG | -0.01 | 0.01 | 0.252 |
|  | SFG - SMA | -0.02 | 0.01 | ***0.003*** |
|  | SFG - PAR | -0.02 | 0.01 | 0.079 |
|  | L pINS/TPJ -PAR | -0.01 | 0.01 | 0.479 |
|  | SC - SC | -0.02 | 0.01 | 0.159 |
| **FS** |  |  |  |  |
|  | PFC - PAR | 0 | 0.01 | 0.909 |
|  | SFG -SFG | -0.01 | 0.01 | 0.489 |
|  | SFG - SMA | 0 | 0.01 | 0.93 |
|  | SFG - PAR | 0 | 0.01 | 0.99 |
|  | L pINS/TPJ -PAR | 0 | 0.01 | 0.717 |
|  | SC - SC | 0 | 0.01 | 0.836 |
| **HAR** |  |  |  |  |
|  | PFC - PAR | -0.03 | 0.01 | ***<0.000*** |
|  | SFG -SFG | -0.02 | 0.01 | ***0.026*** |
|  | SFG - SMA | -0.03 | 0.01 | ***<0.000*** |
|  | SFG - PAR | -0.03 | 0.01 | ***0.004*** |
|  | L pINS/TPJ -PAR | -0.02 | 0.01 | 0.101 |
|  | SC - SC | -0.02 | 0.01 | 0.069 |
| **NAS** |  |  |  |  |
|  | HE | -0.21 | 0.08 | ***0.008*** |
|  | FS | -0.1 | 0.08 | 0.242 |
|  | HAR | -0.41 | 0.07 | ***<0.000*** |

*Health Engagement (HE), Family Systems (FS), and Health and Adult Relationship (HAR) represent 3 factors summarizing 10 health and socioemotional support measures. Outcome column indicates the factor used in linear regressions; Predictor column indicates variables tested for associations with outcomes, with associated regression parameter estimates, error, and p values.*

| **Supplemental Table 5.** Pearson correlation of connectivity and motion for each significant network pair | | | | | | |
| --- | --- | --- | --- | --- | --- | --- |
|  | Translational motion (mm) | | | Rotational motion (degrees) | | |
|  | *r* |  | *p-value* | *r* |  | *p-value* |
| PFC - PAR | -0.049 |  | 0.599 | -0.14 |  | 0.130 |
| SFG - SFG | -0.039 |  | 0.676 | -0.141 |  | 0.127 |
| SFG - SMA | -0.134 |  | 0.147 | -0.240 |  | 0.009* |
| SFG - PAR | -0.110 |  | 0.236 | -0.103 |  | 0.266 |
| pINS/TPJ - PAR | -0.067 |  | 0.469 | -0.096 |  | 0.300 |
| SC - SC | -0.057 |  | 0.537 | -0.016 |  | 0.868 |
| Overall WQS connectivity index | 0.008 |  | 0.951 | -0.165 |  | 0.203 |
| *N = 118 for all networks; N = 61 for WQS index* | |  |  |  |  |  |

**Supplemental Table 6.** Comparison of race/ethnicity, child sex, health behavior, maternal age and demographic factors in the study sample and the non-study population

| **Outcome** | | **Study sample** | **Non-study population** | **p value** |
| --- | --- | --- | --- | --- |
| Maternal age, years, M (SD) | | 25.6 (4.5) | 25.7 (5.06) | 0.32 |
| NAS | | -.06 (.833) | .07 (.978) | 0.069 |
| Health Total | | 192.9 (26) | 188.7 (29) | 0.259 |
| Race, ethnicity | |  |  | 0.6 |
|  | African American | 97 (82.2) | 85 (82.5) |  |
|  | Caucasian | 11 (9.3) | 7 (6.8) |  |
|  | Other | 5 (4.2) | 8 (7.8) |  |
|  | Not disclosed | 5 (4.2) | 3 (2.9) |  |
| Relationship status, n (%) | |  |  | 0.69 |
|  | Single | 69 (58.5) | 57 (55.3) |  |
|  | Married/Partnered | 41 (34.7) | 41 (39.8) |  |
|  | Divorced | 1 (0.8) | 0 |  |
|  | Not disclosed | 7 (5.9) | 5 (4.9) |  |
| Annual household income, n (%) | |  |  | 0.57 |
|  | <$10,0000 | 42 (35.6) | 44 (42.7) |  |
|  | $10,000-20,000 | 23 (19.5) | 24 (23.3) |  |
|  | $20,000-30,000 | 19 (16.1) | 12 (11.7) |  |
|  | >$30,000 | 18 (15.3) | 11 (10.7) |  |
|  | Not disclosed | 16 (13.6) | 12 (11.7) |  |
| Maternal education, n (%) | |  |  | 0.33 |
|  | No diploma/No GED | 18 (15.3) | 21 (20.4) |  |
|  | GED/high school diploma | 46 (39.0) | 31 (30.1) |  |
|  | Some college | 43 (36.4) | 37 (35.9) |  |
|  | >=2yr college degree | 6 (5.1) | 11 (610.7) |  |
|  | Not disclosed | 5 (4.2) | 3 (2.9) |  |
| Child sex, n (%) | |  |  | 0.55 |
|  | Female | 48 (40.7) | 46 (44.7) |  |
|  | Male | 70 (59.3) | 57 (55.3) |  |

| **Supplemental Table 7.** Correlations between 5 NAS subscales and connectivity across each significant network pair | | | | | |
| --- | --- | --- | --- | --- | --- |
|  | **Perceived Stress** | **Life Satisfaction** | **Anxiety** | **Worry** | **Depression** |
| PFC - PAR | .396*** | -.267** | .440*** | .426*** | .422*** |
| SFG - SFG | .436*** | -.291** | .328*** | .356*** | .377*** |
| SFG - SMA | .572*** | -.479*** | .457*** | .505*** | .503*** |
| SFG - PAR | .318*** | -.263** | .264** | .236* | .389*** |
| pINS/TPJ - PAR | .357*** | -.219* | .340*** | .368*** | .237* |
| SC - SC | .336*** | -0.078 | .284** | .306*** | .286** |
| ****p*<.001, ***p<*.01, **p*<.05 | | | | |  |

**Supplemental Table 8.** Summary of original and newly-added health behavior items and their hypothesized factors adapted from ^26^.

| **Item #** | **Item label** | **Factor** |
| --- | --- | --- |
| **1** | Limit amount of fat in diet | DIET |
| **2** | Have 5 or more alcoholic drinks per day (R) | SUBSTANCE |
| **3** | Discuss health concerns with my doctor | MEDICAL |
| **4** | Exercise vigorously | EXERCISE |
| **5** | Avoid secondhand smoke | SUBSTANCE |
| **6** | Sleep 7-8 hours a night | SLEEP |
| **7** | Eat non-fat dairy products | DIET |
| **8** | Obtain regular doctor check-ups | MEDICAL |
| **9** | Do exercises that are good for me | EXERCISE |
| **10** | Limit how much sugar I eat | DIET |
| **11** | Eat vegetables every day | DIET |
| **12** | Avoid using tobacco products | SUBSTANCE |
| **13** | Go for regular walks | EXERCISE |
| **14** | Eat healthy food | DIET |
| **15** | Limit my intake of alcohol | SUBSTANCE |
| **16** | Limit amount of salt in my diet | DIET |
| **17** | Do physical exercises I enjoy | EXERCISE |
| **18** | Eat a balanced diet | DIET |
| **19** | Smoke cigarettes daily (R) | SUBSTANCE |
| **20** | Exercise until I am breathing heavily | EXERCISE |
| **21** | Take vitamins daily | DIET |
| **22** | Limit the amount of sweets in my diet | DIET |
| **23** | Eat fast food (R) | DIET |
| **24** | Drink 4 or more glasses of water per day | DIET |
| **25** | Pay attention to the number of calories in the foods I eat | MEDICAL |
| **26** | Go to see a dentist every 6 months | MEDICAL |
| **27** | Have contact with cigarette smoke (R) | SUBSTANCE |
| **28** | Figure out from labels what foods are good for me | DIET |
| **29** | Get adequate sleep every night | SLEEP |
| **30** | Avoid exercising (R) | EXERCISE |
| **31** | Smoke cigarettes excessively (R) | SUBSTANCE |
| **32** | Eat junk food (R) | DIET |
| **33** | Drink alcohol until intoxicated (R) | SUBSTANCE |
| **34** | Seek health care when needed | MEDICAL |
| **35** | Make sure you are physically active | EXERCISE |
| **36** | Not get enough sleep and rest | SLEEP |
| **37** | Drink alcohol excessively (R) | SUBSTANCE |
| **38** | Seek health information | MEDICAL |
| **39** | Read food labels to see what I am eating | DIET |
| **40** | Feel rested and refreshed | SLEEP |
| **41** | Drink coffee* (R) | DIET |
| **42** | Drink soda pop* (R) | DIET |
| **43** | Try to get 6 or more hours of sleep* | SLEEP |
| **44** | Go to the same dentist office for all my dental care* | MEDICAL |
| **45** | Instead of riding an escalator/elevator, take the stairs* | EXERCISE |
| * indicated newly added item. (R) = reverse-scored. We removed items from the original Health Behavior questionnaire developed by Jackson (2006) that may have had low validity in our sample, including being stigmatizing, threatening, or making participants unconformable. We also removed items to shorten the overall length of the questionnaire and reduce participant burden. The items that were removed were: Use drugs to get high, Limit amount of red meat in diet, Perform stretching exercises, Have physically active home life, Report persistent symptoms to doctor, Have your blood pressure checked regularly, Use recreational drugs to relax, Ignore persistent physical symptoms, Eat whole grain foods, Get daily aerobic exercises, Eat foods high in fiber, Consume enough calcium, and Avoid regular doctor checkups. | | |

**Supplemental Table 9.** The inter-subscale correlations were moderate, consistent with the estimates reported by Jackson:

|  | **Diet** | **Exercise** | **Medical** | **Substance Use** |
| --- | --- | --- | --- | --- |
| **Exercise** | .53*** |  |  |  |
| **Medical** | .43*** | .34*** |  |  |
| **Sleep** | .35*** | .33*** | .41*** |  |
| **Substance Use** | .42*** | .19** | .28*** | .30*** |
| ****p*<.001, ***p<*.01 | | | | |

**Supplemental Table 10.** Confirmatory Factor Analysis in Mplus vs. 7.2 using WLSMV estimation appropriate for ordinal scales indicated that the items loaded onto a five-factor model consistent with hypothesized factors

| **Item #** | **Item label** | **Factor** | **Item loading on factor in 5 factor model using WLSMV estimation** | | | |
| --- | --- | --- | --- | --- | --- | --- |
|  |  |  | B | SE | β | *p* |
| **1** | Limit amount of fat in diet | DIET | 0.57 | 0.048 | 0.57 | <0.001 |
| **7** | Eat non-fat dairy products | DIET | 0.301 | 0.064 | 0.301 | <0.001 |
| **10** | Limit how much sugar I eat | DIET | 0.781 | 0.03 | 0.781 | <0.001 |
| **11** | Eat vegetables every day | DIET | 0.565 | 0.05 | 0.565 | <0.001 |
| **14** | Eat healthy food | DIET | 0.721 | 0.037 | 0.721 | <0.001 |
| **16** | Limit amount of salt in my diet | DIET | 0.73 | 0.037 | 0.73 | <0.001 |
| **18** | Eat a balanced diet | DIET | 0.807 | 0.027 | 0.807 | <0.001 |
| **21** | Take vitamins daily | DIET | 0.387 | 0.072 | 0.387 | <0.001 |
| **22** | Limit the amount of sweets in my diet | DIET | 0.802 | 0.027 | 0.802 | <0.001 |
| **23** | Eat fast food | DIET | 0.347 | 0.064 | 0.347 | <0.001 |
| **24** | Drink 4 or more glasses of water per day | DIET | 0.538 | 0.057 | 0.538 | <0.001 |
| **25** | Pay attention to the number of calories in the foods I eat | DIET | 0.591 | 0.047 | 0.591 | <0.001 |
| **28** | Figure out from labels what foods are good for me | DIET | 0.653 | 0.041 | 0.653 | <0.001 |
| **32** | Eat junk food | DIET | 0.412 | 0.06 | 0.412 | <0.001 |
| **39** | Read food labels to see what I am eating | DIET | 0.672 | 0.041 | 0.672 | <0.001 |
| **41** | Drink coffee* | DIET | 0.116 | 0.068 | 0.116 | 0.091 |
| **42** | Drink soda pop* | DIET | 0.334 | 0.059 | 0.334 | <0.001 |
| **4** | Exercise vigorously | EXERCISE | 0.589 | 0.052 | 0.589 | <0.001 |
| **9** | Do exercises that are good for me | EXERCISE | 0.752 | 0.038 | 0.752 | <0.001 |
| **13** | Go for regular walks | EXERCISE | 0.654 | 0.05 | 0.654 | <0.001 |
| **17** | Do physical exercises I enjoy | EXERCISE | 0.925 | 0.023 | 0.925 | <0.001 |
| **20** | Exercise until I am breathing heavily | EXERCISE | 0.254 | 0.073 | 0.254 | 0.001 |
| **30** | Avoid exercising | EXERCISE | 0.506 | 0.055 | 0.506 | <0.001 |
| **35** | Make sure you are physically active | EXERCISE | 0.77 | 0.048 | 0.77 | <0.001 |
| **45** | Instead of riding an escalator/elevator, take the stairs* | EXERCISE | 0.525 | 0.057 | 0.525 | <0.001 |
| **3** | Discuss health concerns with my doctor | MEDICAL | 0.554 | 0.072 | 0.554 | <0.001 |
| **8** | Obtain regular doctor check-ups | MEDICAL | 0.6 | 0.068 | 0.6 | <0.001 |
| **26** | Go to see a dentist every 6 months | MEDICAL | 0.499 | 0.069 | 0.499 | <0.001 |
| **34** | Seek health care when needed | MEDICAL | 0.643 | 0.076 | 0.643 | <0.001 |
| **38** | Seek health information | MEDICAL | 0.805 | 0.058 | 0.805 | <0.001 |
| **44** | Go to the same dentist office for all my dental care* | MEDICAL | 0.472 | 0.076 | 0.472 | <0.001 |
| **6** | Sleep 7-8 hours a night | SLEEP | 0.809 | 0.028 | 0.809 | <0.001 |
| **29** | Get adequate sleep every night | SLEEP | 0.932 | 0.021 | 0.932 | <0.001 |
| **36** | Not get enough sleep and rest | SLEEP | 0.497 | 0.051 | 0.497 | <0.001 |
| **40** | Feel rested and refreshed | SLEEP | 0.829 | 0.03 | 0.829 | <0.001 |
| **43** | Try to get 6 or more hours of sleep* | SLEEP | 0.813 | 0.036 | 0.813 | <0.001 |
| **2** | Have 5 or more alcoholic drinks per day | SUBSTANCE | 0.728 | 0.119 | 0.728 | <0.001 |
| **5** | Avoid secondhand smoke | SUBSTANCE | 0.856 | 0.038 | 0.856 | <0.001 |
| **12** | Avoid using tobacco products | SUBSTANCE | 0.805 | 0.043 | 0.805 | <0.001 |
| **15** | Limit my intake of alcohol | SUBSTANCE | 0.446 | 0.101 | 0.446 | <0.001 |
| **19** | Smoke cigarettes daily | SUBSTANCE | 0.947 | 0.04 | 0.947 | <0.001 |
| **27** | Have contact with cigarette smoke | SUBSTANCE | 0.769 | 0.033 | 0.769 | <0.001 |
| **31** | Smoke cigarettes excessively | SUBSTANCE | 0.911 | 0.032 | 0.911 | <0.001 |
| **33** | Drink alcohol until intoxicated | SUBSTANCE | 0.788 | 0.056 | 0.788 | <0.001 |
| **37** | Drink alcohol excessively | SUBSTANCE | 0.843 | 0.097 | 0.843 | <0.001 |
| **Note.** Model fit statistics: CFI=.92, TLI=.91, RMESA=.05, SRMR=.096. Based on modification indices and item conceptual or semantic overlap, the residuals of the following items were set to covary: “Eat junk food” with “Eat fast food” (*r=*0.556, *p<*0.001), “Read food labels to see what I am eating” with “Figure out from labels what foods are good for me” (*r=*0.678, *p<*0.001), “Go to the same dentist office for all my dental care” with “Go to see a dentist every 6 months” (*r=*0.727, *p<*0.001), and “Figure out from labels what foods are good for me” and “Pay attention to the number of calories in the foods I am eating” (*r=*0.549, *p=*0.001). | | | | | | |

**REFERENCES**

1. Radloff, L.S. The CES-D Scale: A Self-Report Depression Scale for Research in the General Population. *Applied psychological measurement* **1**, 385-401 (1977).

2. Spielberger, C.D. *State-trait anxiety inventory : a comprehensive bibliography*, (Consulting Psychologists Press, Palo Alto, CA, 1984).

3. Meyer, T.J., Miller, M.L., Metzger, R.L. & Borkovec, T.D. Development and validation of the Penn State Worry Questionnaire. *Behav Res Ther* **28**, 487-495 (1990).

4. Cohen, S., Kamarck, T. & Mermelstein, R. A global measure of perceived stress. *J Health Soc Behav* **24**, 385-396 (1983).

5. Diener, E., Emmons, R.A., Larsen, R.J. & Griffin, S. The satisfaction with life scale. *Journal of Personality Assessment* **49**, 71-75 (1985).

6. Muthén, L.K. & Muthén, B.O. *Mplus User’s Guide*, (Muthén & Muthén, Los Angeles, CA, 2014).

7. de Weerth, C. & Buitelaar, J.K. Physiological stress reactivity in human pregnancy--a review. *Neurosci Biobehav Rev* **29**, 295-312 (2005).

8. Kammerer, M., Adams, D., Castelberg Bv, B.V. & Glover, V. Pregnant women become insensitive to cold stress. *BMC Pregnancy Childbirth* **2**, 8 (2002).

9. Wadhwa, P.D., Sandman, C.A., Chicz-DeMet, A. & Porto, M. Placental CRH modulates maternal pituitary adrenal function in human pregnancy. *Ann N Y Acad Sci* **814**, 276-281 (1997).

10. Oswald, L.M.*, et al.* Relationship between cortisol responses to stress and personality. *Neuropsychopharmacology* **31**, 1583-1591 (2006).

11. Ruttle, P.L., Maslowsky, J., Armstrong, J.M., Burk, L.R. & Essex, M.J. Longitudinal associations between diurnal cortisol slope and alcohol use across adolescence: a seven-year prospective study. *Psychoneuroendocrinology* **56**, 23-28 (2015).

12. Wirth, M.M., Scherer, S.M., Hoks, R.M. & Abercrombie, H.C. The effect of cortisol on emotional responses depends on order of cortisol and placebo administration in a within-subject design. *Psychoneuroendocrinology* **36**, 945-954 (2011).

13. Bibbey, A., Carroll, D., Roseboom, T.J., Phillips, A.C. & de Rooij, S.R. Personality and physiological reactions to acute psychological stress. *Int J Psychophysiol* **90**, 28-36 (2013).

14. Burke, H.M., Davis, M.C., Otte, C. & Mohr, D.C. Depression and cortisol responses to psychological stress: A meta-analysis. *Psychoneuroendocrinology* **30**, 846-856 (2005).

15. Lovallo, W.R., Cohoon, A.J., Acheson, A., Sorocco, K.H. & Vincent, A.S. Blunted stress reactivity reveals vulnerability to early life adversity in young adults with a family history of alcoholism. *Addiction* (2018).

16. Lovallo, W.R., Farag, N.H., Sorocco, K.H., Cohoon, A.J. & Vincent, A.S. Lifetime adversity leads to blunted stress axis reactivity: studies from the Oklahoma Family Health Patterns Project. *Biol Psychiatry* **71**, 344-349 (2012).

17. Wells, S.*, et al.* Associations of hair cortisol concentration with self-reported measures of stress and mental health-related factors in a pooled database of diverse community samples. *Stress* **17**, 334-342 (2014).

18. Craddock, R.C., James, G.A., Holtzheimer, P.E., 3rd, Hu, X.P. & Mayberg, H.S. A whole brain fMRI atlas generated via spatially constrained spectral clustering. *Hum Brain Mapp* **33**, 1914-1928 (2012).

19. Serag, A.*, et al.* Construction of a consistent high-definition spatio-temporal atlas of the developing brain using adaptive kernel regression. *Neuroimage* **59**, 2255-2265 (2012).

20. van den Heuvel, M., Mandl, R. & Hulshoff Pol, H. Normalized cut group clustering of resting-state FMRI data. *PLoS One* **3**, e2001 (2008).

21. Whitfield-Gabrieli, S. & Nieto-Castanon, A. Conn: a functional connectivity toolbox for correlated and anticorrelated brain networks. *Brain Connect* **2**, 125-141 (2012).

22. Rosvall, M. & Bergstrom, C. Maps of random walks on complex networks reaveal community structure. *PNAS* **105**, 1118-1123 (2008).

23. Eggebrecht, A.T.*, et al.* Joint Attention and Brain Functional Connectivity in Infants and Toddlers. *Cereb Cortex* **27**, 1709-1720 (2017).

24. Moos, R. & Moos, B. *Family Environment Scale Manual: Development, Applications, Research*, (Consulting Psychologist Press, Palo Alto, CA, 1994).

25. Fraley, R.C., Waller, N.G. & Brennan, K.A. An item response theory analysis of self-report measures of adult attachment. *J Pers Soc Psychol* **78**, 350-365 (2000).

26. Jackson, T. Relationships between perceived close social support and health practices within community samples of American women and men. *Journal of Psychology* **140**, 229-246 (2006).

27. Johnson, T.P. Sources of Error in Substance Use Prevalence Surveys. *International scholarly research notices* **2014**, 923290 (2014).

28. Johnson, T.P. & Bowman, P.J. Cross-cultural sources of measurement error in substance use surveys. *Substance use & misuse* **38**, 1447-1490 (2003).

29. Zajacova, A. & Dowd, J.B. Reliability of self-rated health in US adults. *American journal of epidemiology* **174**, 977-983 (2011).

30. Ciric, R.*, et al.* Benchmarking of participant-level confound regression strategies for the control of motion artifact in studies of functional connectivity. *Neuroimage* **154**, 174-187 (2017).

31. Blazejewska, A.I.*, et al.* 3D in utero quantification of T2* relaxation times in human fetal brain tissues for age optimized structural and functional MRI. *Magn Reson Med* **78**, 909-916 (2017).

32. Vasylechko, S.*, et al.* T2* relaxometry of fetal brain at 1.5 Tesla using a motion tolerant method. *Magn Reson Med* **73**, 1795-1802 (2015).

33. Jakab, A.*, et al.* Fetal functional imaging portrays heterogeneous development of emerging human brain networks. *Front Hum Neurosci* **8**, 852 (2014).

34. Turk, E.*, et al.* Functional brain connectomic blueprint is apparent in the second and third trimester of pregnancy. *J. Neurosci.* (2019).

35. Gholipour, A.*, et al.* A normative spatiotemporal MRI atlas of the fetal brain for automatic segmentation and analysis of early brain growth. *Scientific reports* **7**, 476 (2017).

36. Wright, R.*, et al.* Construction of a fetal spatio-temporal cortical surface atlas from in utero MRI: Application of spectral surface matching. *Neuroimage* **120**, 467-480 (2015).

37. Anderson, A.L. & Thomason, M.E. Functional plasticity before the cradle: A review of neural functional imaging in the human fetus. *Neurosci. Biobehav. Rev.* **37**, 2220-2232 (2013).

38. van den Heuvel, M.I. & Thomason, M.E. Functional Connectivity of the Human Brain in Utero. *Trends Cogn Sci* **20**, 931-939 (2016).

39. Schopf, V., Kasprian, G. & Prayer, D. Functional imaging in the fetus. *Top Magn Reson Imaging* **22**, 113-118 (2011).

40. Thomason, M.*, et al.* Intrinsic functional brain architecture derived from graph theoretical analysis in the human fetus. *PLoS One* (2014).

41. Thomason, M.*, et al.* Cross-hemispheric functional connectivity in the human fetal brain. *Science translational medicine* **5**(2013).

42. Wheelock, M.D.*, et al.* Sex differences in functional connectivity during fetal brain development. *Developmental cognitive neuroscience* **36**, 100632 (2019).

43. Buss, C., Entringer, S. & Wadhwa, P.D. Fetal programming of brain development: intrauterine stress and susceptibility to psychopathology. *Sci Signal* **5**, pt7 (2012).

44. Entringer, S., Buss, C. & Wadhwa, P.D. Prenatal stress and developmental programming of human health and disease risk: concepts and integration of empirical findings. *Current opinion in endocrinology, diabetes, and obesity* **17**, 507-516 (2010).

45. Sandman, C.A., Davis, E.P., Buss, C. & Glynn, L.M. Prenatal programming of human neurological function. *Int J Pept* **2011**, 837596 (2011).

46. Moog, N.K.*, et al.* Influence of maternal thyroid hormones during gestation on fetal brain development. *Neuroscience* **342**, 68-100 (2017).
